# Toward a theory of coactivation patterns in excitable neural networks

**DOI:** 10.1371/journal.pcbi.1006084

**Published:** 2018-04-09

**Authors:** Arnaud Messé, Marc-Thorsten Hütt, Claus C. Hilgetag

**Affiliations:** 1 Institute of Computational Neuroscience, University Medical Center Eppendorf, Hamburg University, Hamburg, Germany; 2 Department of Life Sciences and Chemistry, Jacobs University, Bremen, Germany; 3 Department of Health Sciences, Boston University, Boston, Massachusetts, United States of America; Ghent University, BELGIUM

## Abstract

The relationship between the structural connectivity (SC) and functional connectivity (FC) of neural systems is of central importance in brain network science. It is an open question, however, how the SC-FC relationship depends on specific topological features of brain networks or the models used for describing neural dynamics. Using a basic but general model of discrete excitable units that follow a susceptible—excited—refractory activity cycle (SER model), we here analyze how the network activity patterns underlying functional connectivity are shaped by the characteristic topological features of the network. We develop an analytical framework for describing the contribution of essential topological elements, such as common inputs and pacemakers, to the coactivation of nodes, and demonstrate the validity of the approach by comparison of the analytical predictions with numerical simulations of various exemplar networks. The present analytic framework may serve as an initial step for the mechanistic understanding of the contributions of brain network topology to brain dynamics.

## Author summary

Functional connectivity, as reflected in the statistical dependencies of distributed activity, is widely used to probe the organization of complex systems such as the brain. While this measure has been helpful for characterizing brain states and highlighting alterations of brain dynamics in various diseases, the mechanisms underlying the generation of FC patterns remain poorly understood. One prominent factor shaping FC is the underlying neural network structure. Using a minimalist model of excitation, we investigate how the topology of excitable neural networks contributes to FC. Specifically, we show that FC can be analytically predicted from the way in which the nodes are embedded in the network and how they are related to basic self-organizing units of excitable dynamics, particularly, short pacemaker cycles. These insights are a step towards a mechanistic understanding of the activation patterns of complex neural networks.

## Introduction

The network perspective has become a powerful and central approach for representing and analyzing complex biological systems, spanning from the study of interacting genes to neuronal assemblies [1]. Particularly for brain networks, a fundamental challenge is to understand the relationship between the network organization (or topology) of the structural connectivity and network activity or dynamics, as reflected by the network’s functional connectivity [2].

For more than a decade, increasingly sophisticated computational neuroscience approaches have been used to model the activity patterns of brain networks based on their characteristic network connectivity [3–7]. These approaches have used a variety of highly diverse node models, ranging from detailed biophysical models, eg, [8], neural mass models summarizing the properties of neuronal populations [9], to more abstract and phenomenological models, such as the Kuramoto model [10, 11], as well as stylized simple discrete models of neural excitation [12]. Intriguingly, the choice of the specific computational model does not seem to crucially affect the resulting global patterns of functional connectivity, as highly diverse models applied on the same network may result in very similar fits of the empirical FC [13, 14]. This finding suggests that the crucial aspect in producing functional connectivity may not be the specific local models, but the characteristic topology of the underlying SC. Ubiquitous topological features of brain networks are, for instance, modules (formed by nodes that are more frequently connected among each other than to the rest of the network) and hubs (central nodes that have more connections than average network nodes) [15, 16].

Our present goal is to systematically explore key topological contributions to SC-FC relationships, and develop a mechanistic understanding of the involved elementary processes: How do excitable dynamics translate specific topological patterns into systematic coactivations of nodes or functional connectivity? While previous studies have demonstrated that network topology matters in determining the network activity patterns, they have not yet provided a universal framework of the SC-FC relationship. Several studies have used sets of intuitive topological rules, including the topological overlap, paths distribution, as well as branching and convergence of projections, for predicting FC [17–19]. While these predictions were comparable to more sophisticated computational models in successfully predicting the empirical FC, the predictions were still far from perfect, leaving room for further improvements as well as an analytic understanding of the SC-FC relationship.

As we strive for a mechanistic understanding and an analytical description of how SC-FC relationships change with network architecture, we study this question in a minimal, deterministic model of excitable dynamics. Subsequent investigations will then analyze the relevance of our findings for more general dynamical regimes. Here, we used the stylized dynamics of the discrete excitable SER model [20]. The letters S-E-R denote the basic node behavior of susceptible (S) nodes becoming excited (E) by excited neighbors, then refractory (R), before turning once again susceptible, in discrete time steps. What the SER model offers is a detailed mechanistic understanding of how topology regulates particular contributions to the coactivation matrix and in this way determines FC. This minimal model of an excitable system has a rich history in many disciplines, ranging from the propagation of forest-fires [21, 22], the spread of epidemics [23, 24], to neuronal dynamics [25, 26]. Using a similar model setup we have shown that the distribution pattern of excitations is regulated by the connectivity as well as by the rate of spontaneous excitations [27]. An increase in each of these two quantities leads to a sudden increase in the excitation density accompanied by a drastic change in the distribution pattern from a collective, synchronous firing of a large number of nodes in the graph to more local, long-lasting and propagating excitation patterns. More recently, and focusing particularly on the topology of excitable networks, we have shown that SC-FC relationships strongly and non-trivially depend on neural network topology [28–30]. Qualitatively speaking, modularity is associated with high positive correlations between SC and FC, while a broad degree distribution, or low network density, may lead to negative SC-FC correlations. The mechanism behind this finding is as follows. High connectivity is associated with an elevated excitation density. Locally high connectivity (that is, within a module) results in a statistically higher number of excitations among nodes within the same module and, as a consequence, systematically higher coactivations of nodes in the same module. This positive contribution to the correlation between SC and FC tends to ‘overwrite’ the typically negative correlations arising from suppressed coactivations of linked nodes due to sequential excitation [28]. Moreover, in [31] a first evidence for a dependence of dynamical features on the initial conditions and the potential relevance of triangles was provided. The discrete SER model, thus, provides us with clear hypotheses of how SC is translated into FC.

In the present work, we present a computational framework for analytically predicting patterns of functional connectivity from excitable dynamics running on an arbitrary network architecture. Our theoretical framework should be capable of addressing two main questions: (1) Why do different network topologies show systematically different levels of SC-FC relationships? (2) How do specific subsets of initial conditions suppress or enhance different topological features and hence lead to systematically altered coactivation matrices and, as a consequence, alterations in SC-FC relationships? After illustrating the operation of the SER model with some introductory examples, we present potential analytical predictions with increasing realism. Subsequently, we explore to which extent these predictions can reproduce simulated patterns of FC across different topological network configurations.

## Results

**Fig 1:**
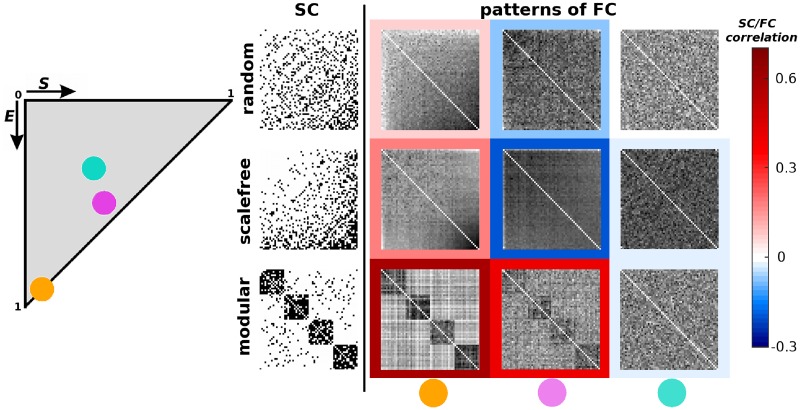
Examples of FC patterns in the deterministic SER model. (Left) State space of all possible initial conditions. (Right) Exemplar structural connectivity (first column), and associated patterns of coactivation (next columns) for different initial conditions as represented by colored disks. Each row represents a typical network topology, specifically, random (first row), scale-free (middle row) and modular (last row).

The topological structure of a network is represented by the adjacency matrix *A*, where *A*_*ij*_ = 1, if nodes *i* and *j* are connected, 0 otherwise. Here we consider only undirected networks. In a simplifying approach, we consider functional connectivity to be the coactivation of nodes derived from excitation patterns of the deterministic SER model on a given graph. In the following investigations, *T* denotes the probability of having a node in the T state in the initial conditions (or put differently, the proportion of nodes in the T state, at first), *T* = Pr(node state = T) with T ∈ {S, E, R}. The predictive value of the different analytical proposals, including SC itself, was investigated by computing the (Pearson) correlation as well as the mean signed difference between the simulated and predicted FCs. For illustrative purposes, Fig 1 shows a set of functional connectivity patterns resulting from the SER dynamics starting at different initial conditions. Strikingly, FC is not only shaped by the network topology, but also strongly by the initial conditions of the SER model. For example, negative SC-FC correlations as observed in scale-free graphs can turn into positive correlations, depending on the initial conditions (Fig 1). Throughout the manuscript, unless otherwise stated, we explore the effect of initial conditions such that *E* is varied between 0 and 1, while *R* = *S* = (1 − *E*)/2. See Materials and Methods section for further details.

### Initial steps: A purely topological perspective

Let us consider a pair of nodes (*i*, *j*). The most elementary contribution to coactivation is that a common neighbor jointly activates the two nodes *i* and *j*. This first simple consideration already points to the fact that the number of common neighbors of two nodes could predict functional connectivity. Thus, we obtain a first prediction for the coactivation of nodes *i* and *j*, denoted here TO:

$$\begin{array}{c} TO_{ij}=\left|N_{ij}\right|=\sum_{k}A_{ik}A_{jk}, \end{array}$$ (1)

where $N_{ij}$ denotes the set of common neighbors of the pair (*i*, *j*). This quantity is better known by its normalized version, the matching index or topological overlap [32]. However, this quantity is not able to predict the potential effect of the initial conditions, as no dependence on the probabilities of states is incorporated. In the following section we describe how the exact pattern of triangles around a pair of nodes (*i*, *j*) can generate specific patterns of coactivations. Furthermore, we show that a striking dependence of coactivations on the statistics of initial conditions is instigated through the probability of initializing a triangle as a pacemaker.

### Macroscopic prediction

Typically in the SER simulations, starting from random initial conditions, after a short transient all nodes settle into a period-3 oscillation (except for very sparse graphs). The dynamics, thus, typically partition the nodes of a graph into three cohorts: those jointly active at time *t*, at time *t* + 1 and at time *t* + 2, respectively. The contribution of a single run to the coactivation matrix is, therefore, almost completely determined by the initial conditions, rather than by the network architecture. When accumulating information over a large number of runs, the pattern of coactivations becomes governed by topological features of the graph. In this deterministic setting of the SER model, the drivers of the dynamics are autonomously oscillating triangles serving as pacemakers [28]. A *pacemaker* corresponds to an isolated triangle initialized with any permutation of the three states S, E and R, displaying stable oscillations that cannot be disrupted by noise or surrounding influences when embedded in larger networks. It is a decisive advantage of this deterministic cellular automaton model that it allows for enumerating the full state space of such network motifs.

**Fig 2:**
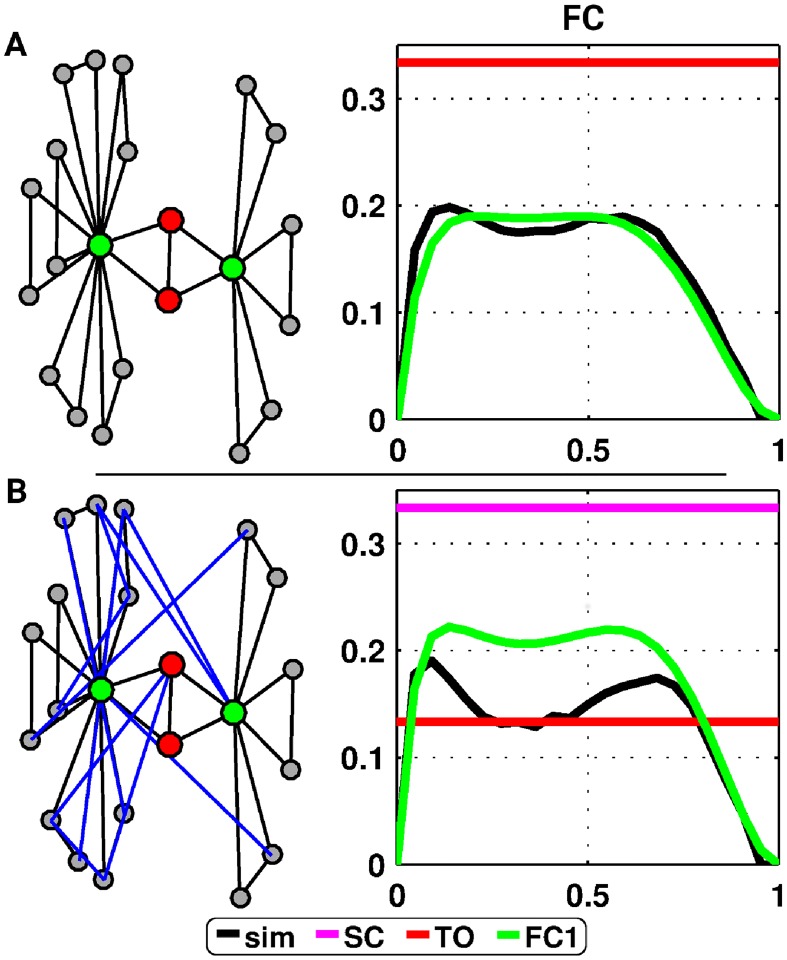
Prediction of the FC patterns in the deterministic SER model for toy examples. (A) Toy structural connectivity network with a pair of nodes in red and their common neighbors in green (top), and associated patterns of coactivation as a function of the initial percentage of excited nodes (bottom). Colors code for the different predictions (magenta, red and green for the prediction from SC, TO—Eq 1 and FC1—Eq 5, respectively, black codes for the simulated FC). (B) Same as (A) with additional links (in blue) added randomly in the original graph.

As an illustration of this general approach, we first consider a small toy network model consisting of the pair of nodes under consideration, a number of common neighbors and *independent* triangles around each of these common neighbors (Fig 2A). We assume that, after a short transient, the system settles into a period-3 oscillation (which corresponds to assuming that the graph has a large enough number of triangles and the initial conditions contain non-zero numbers of nodes in the S, E and R states). For each initial condition, the pattern of coactivations in the deterministic SER model is then essentially a consequence of the two different possible triangle usages: active pacemakers (i.e., triangles initialized as some permutation of S, E, R and, thus, producing cyclic activity) and passive elements driven by other pacemakers (i.e., triangles initialized in any other way, which autonomously would settle into an all-susceptible state, but are typically excited by excitations coming from other parts of the network). Our hypothesis is that, in order to simultaneously activate two nodes *i* and *j*, they must (1) be not part of an active pacemaker and (2) have at least one of their common neighbors part of an active pacemaker.

The derivation of the predictions is inspired by mean-field approaches classically employed in epidemic models [33]. The dynamics of the nodes is characterized at the population level per state, which considerably reduces the dimensionality of the system. For example, the probability of finding a pair of nodes in the SR state in the initial conditions simply reduces to the product of the marginal probabilities, that is, *SR*. In the following section we formulate a first prediction for the case of non-overlapping triangles (among nodes *i* and *j*, as well as among their common neighbors). The probability that the pair (*i*, *j*) and one of their common neighbors form an active pacemaker is:

$$\begin{array}{c} {△}_{ij}=2A_{ij}[SR\left(1-{\left(1-E\right)}^{|N_{ij}|}\right)+SE\left(1-{\left(1-R\right)}^{|N_{ij}|}\right)+RE\left(1-{\left(1-S\right)}^{|N_{ij}|}\right)], \end{array}$$ (2)

The set of common neighbors of nodes *i* and *j* is characterized by the number of triangles around each of these neighbors (not including the one formed with nodes *i* and *j*). Let *c*_*ijk*_ be the number of triangles around the *k*th common neighbor of the pair (*i*, *j*), this quantity corresponds to the unnormalized clustering coefficient of *k* minus one, if (*i*, *j*) are connected

$$\begin{array}{c} c_{ijk}=C_{k}d_{k}\left(d_{k}-1\right)/2-A_{ij}, \end{array}$$ (3)

where *C*_*k*_ (resp. *d*_*k*_) is the clustering coefficient (resp. the degree) of node *k*. Then the probability that this neighbor is not part of an active pacemaker is

▽ijk=S(1-2RE)cijk+E(1-2SR)cijk+R(1-2SE)cijk. (4)

Taking together Eqs 2 and 4, we obtain a prediction for the coactivation of nodes *i* and *j*, named FC1:

FC1ij=(1-△ij)(1-∏k∈Nij▽ijk)/3, (5)

where the factor 1/3 is taking into account the maximum excitation frequency of nodes in the SER dynamics.

The prediction of coactivation probability, Eq 5, does not require the two nodes to be in the same state. When considered in isolation, the two nodes actually have the capacity to synchronize their respective phases. Indicative of such a synchronization are consecutive time steps of a node spent in the S state. In practice (i.e., when embedding such a substructure in a broader network context), the capacity of a pair of nodes to synchronize in such a way is strongly reduced due to other incoming excitations. A further difference between these toy model networks and more general topological situations is that a much wider range of triangle types needs to be taken into account. When considering more realistic (complex) networks, the previous prediction fails to match simulations (Fig 2B). In fact, given the deterministic nature of the model, any triangle used as a pacemaker in the neighborhood of a pair of nodes contributes (non-trivially) to the coactivations. Therefore, we have to enumerate all possible triangle configurations surrounding a pair of nodes.

### Counting triangles

**Fig 3:**
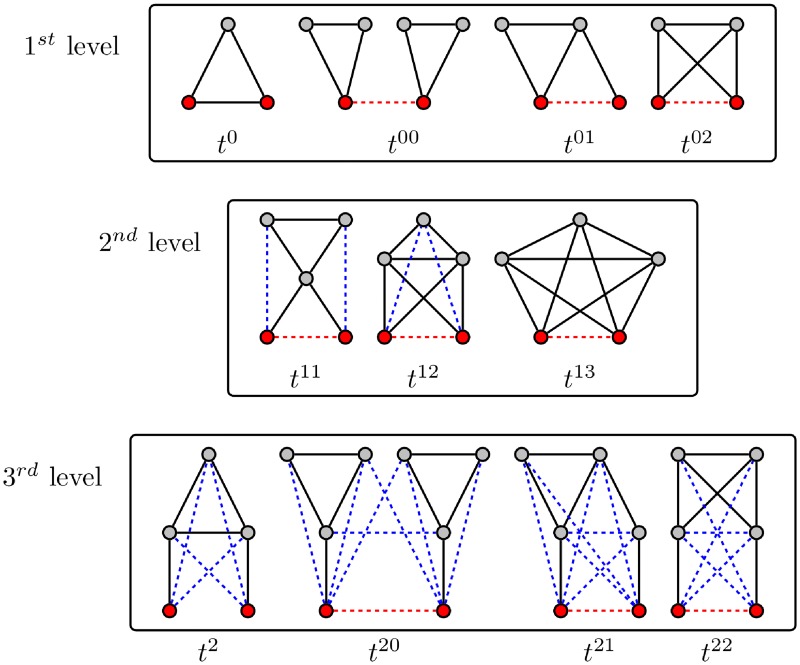
Potential triangle motifs surrounding a pair of nodes. The first row represents the first level of the hierarchy of triangles, with triangles adjacent to at least one node of the pair, while the second row represents the second level with triangles non-adjacent to the pair, but adjacent to at least one common neighbor. The third row represents the third level, where triangles are non-adjacent to the pair and to the set of their common neighbors, but adjacent to at least one neighbor of one node of the pair. Red nodes represent the pair considered, red (resp. blue) dashed edges represent optional edges (resp. potential motifs not considered in the current formalism).

There exists a variety of triangle motifs potentially surrounding a pair of nodes (Fig 3). In the following, we classify such triangles according to their distance to the nodes of interest. First, we have triangles adjacent to the pair which can be characterized as follows, triangles adjacent to:

- *i* **and** *j*: *t*^0^, this quantity corresponds to TO 1 multiplied by the adjacency matrix;
- *i* **or** *j* and **zero** common neighbor: *t*^00^, where $t_{Ij}^{00}$ ($t_{iJ}^{00}$) represent the number of triangles adjacent to *i* (resp. *j*);
- *i* **or** *j* and **one** common neighbor: *t*^01^, where $t_{Ijk}^{01}$ ($t_{iJk}^{01}$) represent the number of triangles adjacent to *i* (resp. *j*) and to the *k*th common neighbor;
- *i* **or** *j* and **two** common neighbors: *t*^02^, where $t_{Ij}^{02}=t_{iJ}^{02}$ represent the number of triangles adjacent to *i* or *j*.

One step further, we have a set of triangles not directly adjacent to (*i*, *j*), but adjacent to their common neighbors; as such we have the set of triangles adjacent to:

- **one** common neighbor: *t*^11^, where $t_{ijk}^{11}$ represent the number of triangles adjacent to the *k*th common neighbor;
- **two** common neighbors: *t*^12^, where $t_{ijl}^{12}$ represent the number of triangles adjacent to the *l*th pair of common neighbors;
- **three** common neighbors: *t*^13^.

Such an arrangement defines an hierarchy of triangles, where the levels are defined by the distance from the pair of nodes. The first level corresponds to the set {*t*^0*k*^} where the average distance is zero, {*t*^1*k*^} represents the second level with an average distance of 1. In this way, we can iteratively define the successive levels of the hierarchy, for instance the third level (Fig 3). However, from a dynamical perspective, higher level triangles have a negligible contribution to FC. Moreover, taking into account such higher order triangles significantly complicates the analytical prediction (see below).

### Microscopic prediction using pacemakers

Once having enumerated all possible triangles surrounding a pair of nodes, we can now in a systematic way enumerate all possible contributions (or non-contributions) of these triangles to FC if used as pacemakers. We introduce here a new notation for easier reading. In particular, a circled arrow around a topological quantity crossed indicates that it is cancelled, meaning that no triangle is used as pacemaker. The orientation of the arrow represents the direction of propagation of the excitation within the triangle (double arrows represent the possibility of excitation running in both ways). Moreover, capital letters are used for indices *i* and/or *j* if a pacemaker is adjacent to them. For example, the above prediction reads as follows:

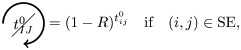

is the probability of having no triangle adjacent to both *i* and *j* (*t*^0^) used as pacemaker, and,

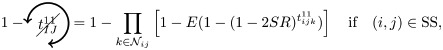

is the probability to have at least one triangle used as a pacemaker adjacent to a common neighbor of the pair (*i*, *j*) and to *i* and *j* at one step distance. Then, when we merge all conditional probabilities, the probability of coactivations, named FC2, reads:

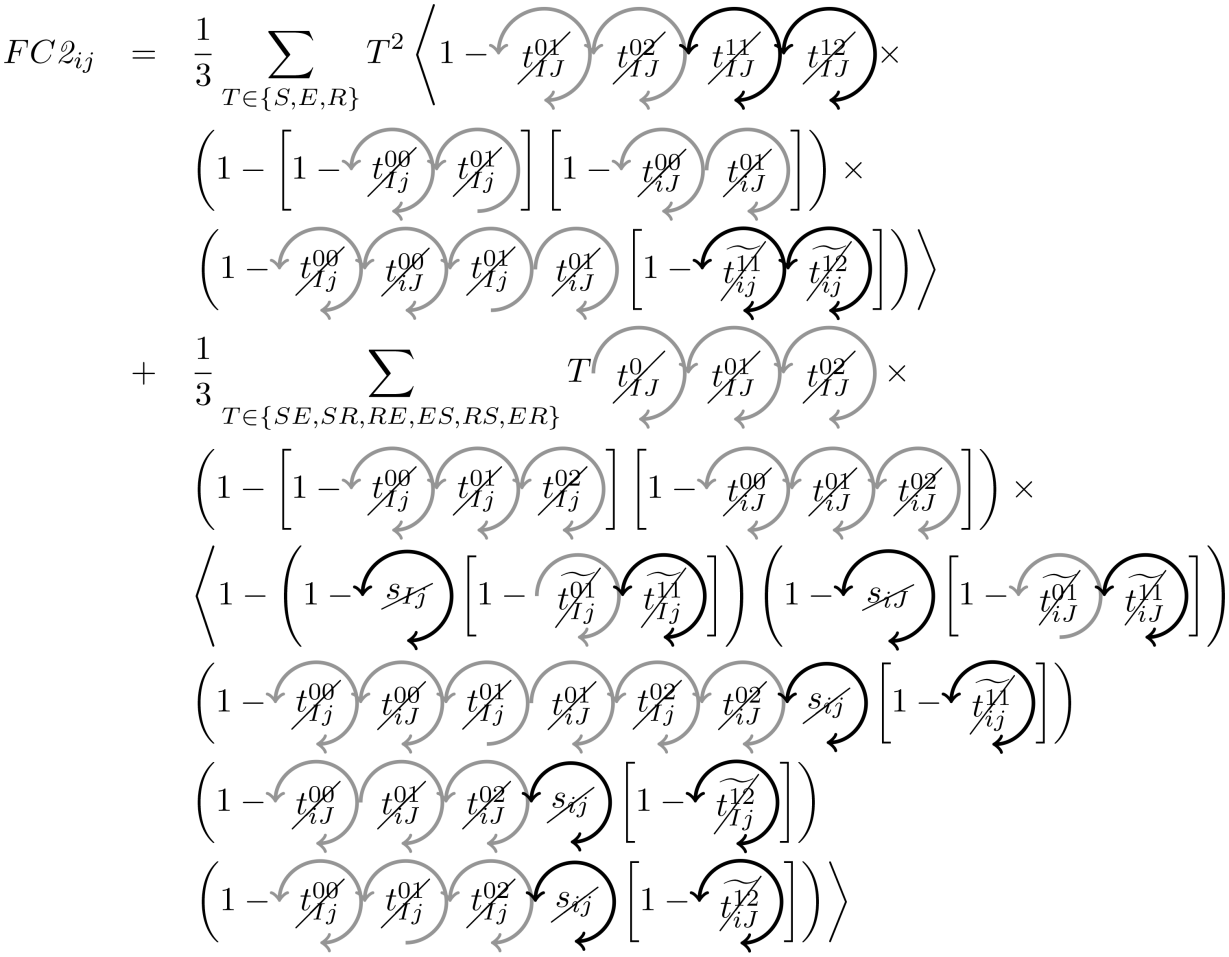
(6)
where greylevel codes for the levels (1*st* gray and 2*nd* black). See Materials and Methods section for further details.

### Relative predictive power

**Fig 4:**
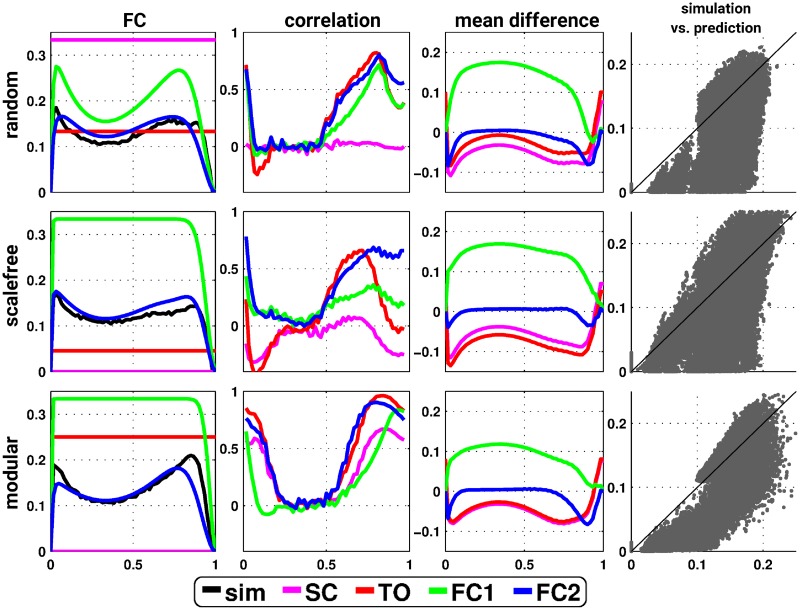
Illustration of the predictive power of the analytical predictions of FC in the deterministic SER model. Example of coactivation (FC) for a randomly selected pair of nodes (first column), correlation (second column) and mean (signed) difference (third column) between simulated and predicted FCs. The plots are a function of the number of nodes initially in the E state and the analytical predictions (magenta, red, green and blue for the prediction from SC, TO—Eq 1, FC1—Eq 5 and FC2—Eq 6, respectively, black codes for the simulated FC). The last column represents the scatter plot of the relation between FC2 (Eq 6) and simulated FC. Each row represents a typical network topology, that is, random (first row), scale-free (middle row) and modular (last row).

We investigated the relative predictive power of the different analytical formulae (including SC, TO, FC1 and FC2) across three typical network topologies (see Materials and Methods section). The formalism based on pacemakers (FC2—Eq 6) in general has the best predictive power, both in terms of correlation and mean difference (Fig 4). FC1 (resp. SC and TO) generally over- (resp. under-) estimated the empirical FC. The predictions appear robust across network realizations as well as across different level of network’s density (S1 Fig). Additionally, we also verified and validated our formalism with generic graphs with a given triangle motifs distribution (S2 Fig).

**Fig 5:**
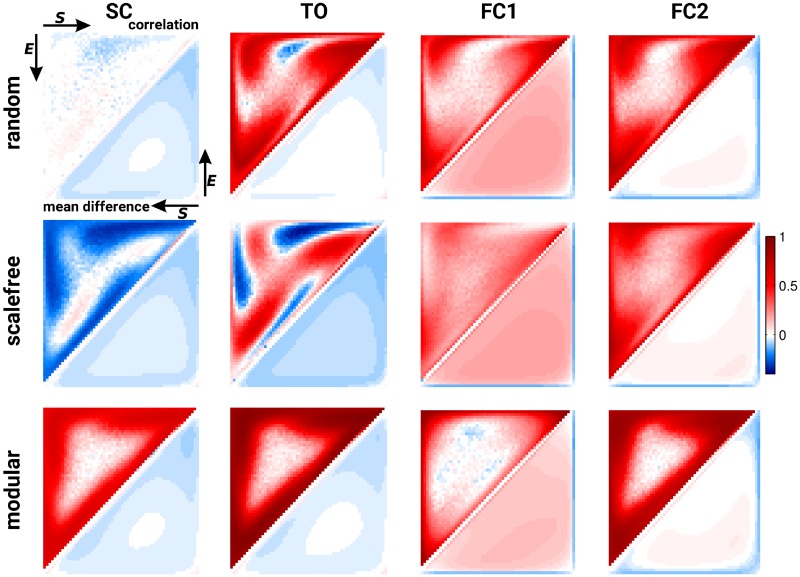
Predictive power of the analytical predictions of FC over the full space of initial conditions in the deterministic SER model. Columns code for the different potential predictors (SC, TO—Eq 1, FC1—Eq 5 and FC2—Eq 6, from left to right) and rows represent typical network topology. For each panel, the upper (resp. lower) triangular parts of the matrices represent the correlation (resp. mean difference) between the simulated FC and its predictor.

Next, we fully explore the space of possible initial conditions. We observed that the correlation between SC and FC is highly specific for the topological properties of the underlying structural network. As previously reported, random networks display no or slightly negative SC-FC correlations (depending on their density), scale-free graphs have moderate negative correlations between SC and FC, and modular networks show strong positive correlations (Fig 5). Additionally, the analytical framework based on pacemakers is able to predict the simulated FC for a relatively wide range and, thus, across almost the full space of possible initial conditions.

## Discussion

In the present study, we developed an analytical framework for predicting the patterns of functional connectivity (that is, the coactivations of nodes) in excitable dynamics on neural networks. As an initial step, we considered a minimal model of neural excitation, the deterministic SER model. The predictions were based on key dynamical ingredients in the deterministic regime of the model, that is, period-3 oscillations and the presence of triangles serving as pacemakers The proposed analytical formula generally predicts the simulated FC across a wide range of initial settings. The framework can be extended to achieve arbitrarily high precision, by including even higher order triangles motifs. However, there are diminishing returns due to the very small contributions of the higher order motifs, which are obtained at a high degree of computational complexity.

This study provides a fully analytic framework for predicting patterns of functional connectivity from the structural topology of networks. Previous work has already demonstrated that it is the network topology, rather than the specific computational model, that shapes FC [13, 14]. While some intuitive rules can be formulated regarding the features of the topology that may be relevant [17], a substantiated mechanistic explanation of the contributions of different network elements to non-stationary activity patterns and FC has so far been lacking. The present framework achieves this goal for the deterministic SER model and allows to predict FC for a wide range of initial conditions with high accuracy.

While the SER model at first glance may appear overly simplistic, it captures the essential steps of the susceptibility, excitation and recovery of neural dynamics and, due to this general nature, can be widely applied in neuroscience. Indeed, variants of this kind of discrete excitable model have been broadly used to simulate neural network dynamics ranging from the interactions of single cells [25, 26, 34] to multi-scale whole brain dynamics [35, 36]. When combined with an appropriate forward model, the discrete excitable model can also be brought to produce realistic approximations of observable neural dynamics, such as in fMRI BOLD signals [12, 30]. Thus, the present framework may also be used for the analytical prediction of empirical functional connectivity derived from large-scale neuroimaging.

At the same time, the discrete excitable model has some methodological advantages over more detailed and complex models of neural dynamics (such as biophysical and mass models). Due to the limited number of its parameters, involving only the statistics of the three states, as well as the absence of further free parameters, the model facilitates a systematic exploration of the initial conditions of excitable networks. Indeed, for suitably small graphs, the model even allows the exhaustive characterization of the complete operating range of a particular network architecture. Empirically, the dependence of dynamical patterns on initial conditions is still largely unexplored, due to the technical challenges of controling such factors. However, there is some evidence showing that neuronal excitability may modulate brain functional connectivity, for example in Alzheimer’s disease [37]. Moreover, the deterministic setting of the model allows the systematic identification of the topological contributions to the coactivation patterns of nodes. For instance, we observed that the predictions work generally fairly well for modular graphs, as in [30]. This is an crucial observation, as virtually all networks in systems biology are modular.

The insights gathered in the present study can be used as a basis for exploring more elaborate models of neural dynamics. In particular, it needs to be explored how the topological predictors of activation patterns identified in the present study can be transposed into more complicated scenarios, such as the stochastic version of the SER model [38] or alternative dynamical models, such as the Fitzhugh-Nagumo model [30]. These next steps will further deepen our mechanistic understanding of how the characteristic topological features of complex brain networks contribute to their global activity patterns and function.

## Materials and methods

### Networks

To investigate the role of topology for the functional connectivity patterns of networks in the SER model, we considered three different types of undirected benchmark graphs: random, scale-free, and modular networks. The random graph was the classical Erdős-Rényi model [39], the scale-free graph was the Barabási-Albert model [40], and the modular graph was a composition of four small random graphs of identical size and with few links among them. The artificial networks had 60 nodes and about 800 links, and were generated with the software package NetworkX [41] as used in [28]. The layouts were generated using a force-directed algorithm [42].

Additionally, we also explored the robustness of the predictions across various network realizations and densities (from 0.1 to 0.6 by step of 0.1). For each density value, we generated 50 synthetic random graphs with 60 nodes, computed the simulated and predicted FCs, and quantified the predictive power of the analytical proposals. Synthetic graphs were generated using the Brain Connectivity Toolbox (https://sites.google.com/site/bctnet/) [43].

### Model

We used a simple three-state cellular automaton model of excitable dynamics, the SER model, representing a stylized biological neuron or neural population [20]. The SER model operates in discrete time and employs the following synchronous update rules, a node in the:

- S → E if at least one neighbor is excited;
- E → R;
- R → S.

This deterministic version was investigated in detail in [28], where, for example, the role of cycles in storing excitations and supporting self-sustained activity was elucidated. The only remaining parameters are the underlying topology of the structural connectivity on which the model runs, and the pattern of initial states.

### Functional connectivity

After appropriate initialization of the deterministic model, the network activity settles into a regular periodic behavior. Therefore, the nodes are divided into distinct groups; nodes are in the same dynamic group when they are simultaneously active. To analyze the pattern of joint excitations, we computed the number of simultaneous excitations for all pairs of nodes. The outcome matrix is the so-called coactivation matrix, a representation of the functional connectivity of the nodes:

$$\begin{array}{c} C_{ij}=\sum_{t}1_{E}\left(x_{i}^{t}\right)1_{E}\left(x_{j}^{t}\right), \end{array}$$

where $x_{i}^{t}$ ∈ {S, E, R} being the state of node *i* at time *t*, and $1_{E}$ the indicator function of state E

$$\begin{array}{c} 1_{E}\left(x_{i}^{t}\right)=\{\begin{array}{c} 1,\;\text{if}\;x_{i}^{t}\in \text{E}\\ 0,\;\text{otherwise} \end{array} \end{array}$$

### Numerical details

In the deterministic SER model, for each network and each initial condition setting, we simulated 5 000 runs of 50 time steps. Unless otherwise stated, the initial conditions were randomly generated, with a probability to set a node into the excited state E between 0 and 1; while the remaining nodes were equipartitioned into susceptible S and refractory R states. FC was summed over runs and normalized by dividing by the product of the number of runs and time steps, scaling FC values between 0 and 1/3. This normalized coactivation matrix was used for all subsequent analyses.

In order to probe the predictive power of the different analytical proposals, including SC itself, we computed the Pearson correlation as well as the mean signed difference between the simulated and predicted FCs. Diagonal elements of matrices were excluded to avoid spurious variations of the prediction. Additionally, SC and TO (the purely topological predictors) were normalized when computing their predictive values, to avoid spurious variations of mean differences with simulated FCs. The normalization was done by dividing them by three times their maximum, effectively scaling the values between 0 and 1/3, as for FC.

### Analytical prediction of FC based on pacemakers

We here enumerate in a systematic way all possible (non-)contributions of the triangles motifs to FC if used as pacemakers. Given the periodic behavior of pacemakers, in the following treatment we only describe the cases where a pair of nodes (*i*, *j*) is in SS or SE states, all others configurations can be deduced in a similar way.

We have to consider two main components. The first one is the set of pacemakers which contribute (or not) systematically to FC, and the other one corresponds to the set of pacemakers which may lead to a systematic contribution to FC providing that the pair (*i*, *j*) synchronizes over time. The systematic (non-)contribution of each triangle motif is as follows:

- *t*^0^ never contributes to FC if at least one triangle is used as a pacemaker which leads to the following criteria:

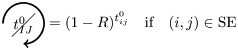
- *t*^00^ can systematically contribute to FC if at least one adjacent triangle is used as a pacemaker at each node, and that these pacemakers are synchronized (i.e. nodes *i* and *j* are in the same initial state):

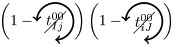

where

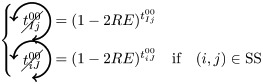

Additionally, if *i* and *j* are in different states (e.g. SE), then we must not have adjacent pacemakers at each node:

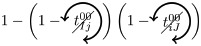
- *t*^01^ can systematically contribute to FC if nodes *i* and *j* are in the same initial state. We have to consider the direction of excitation propagation. If we have one pacemaker adjacent to *i* or *j* and the excitation propagating from the common neighbor to the pair (*i*, *j*), then it will systematically contribute to FC, otherwise we need a least one pacemaker adjacent at both nodes:

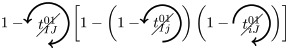

where

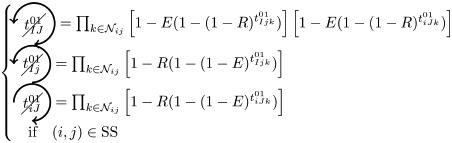

If *i* and *j* are in different states (e.g. SE), then *t*^01^ will never contribute to FC in two cases. The first case is when *i* (or *j*) is adjacent to a pacemaker and *j* (or *i*) is also adjacent to this pacemaker, but at a distance of one (step). For example, if (*i*, *j*) ∈ SE and *i* is adjacent to one pacemaker where the common neighbor is in *R* state, then *j* will be enslaved by the activity of this pacemaker. The second case corresponds to having two independent pacemakers adjacent to *i* and *j*. Additionally, the set (*i*, *j*, *k*) must not form a pacemaker if *A*_*ij*_ = 1:

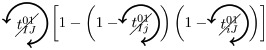

where

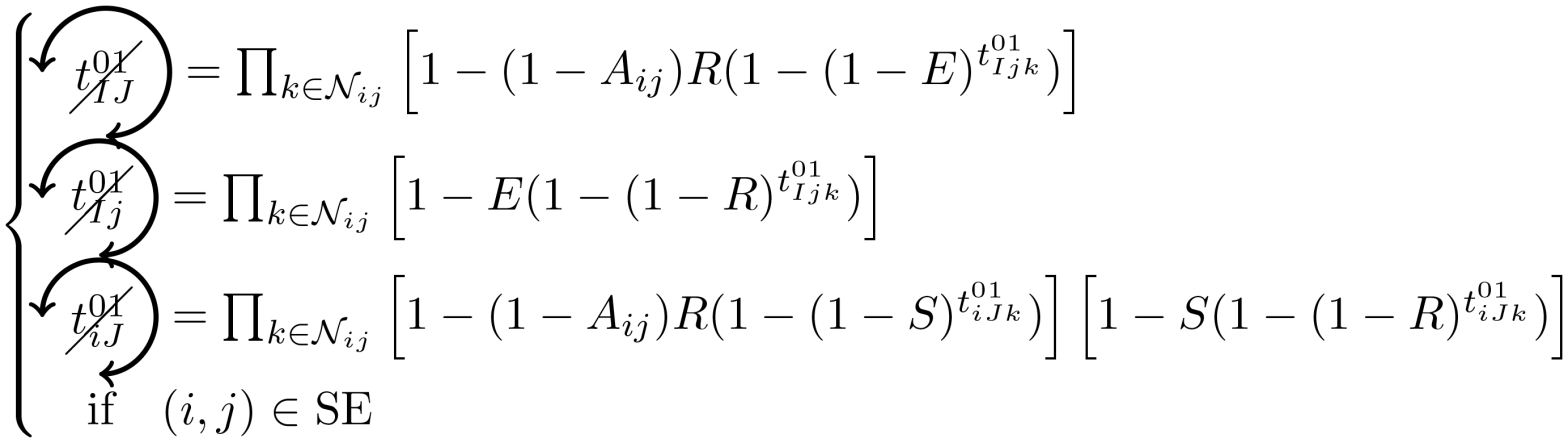
- *t*^02^ will always contribute to FC, if at least one of the triangles is used as a pacemaker and the two nodes are in the same initial state

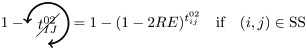

If *i* and *j* are in different states (e.g. SE), then we must not have pacemakers adjacent to both nodes or independent pacemakers adjacent to each node:

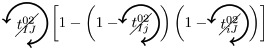

where

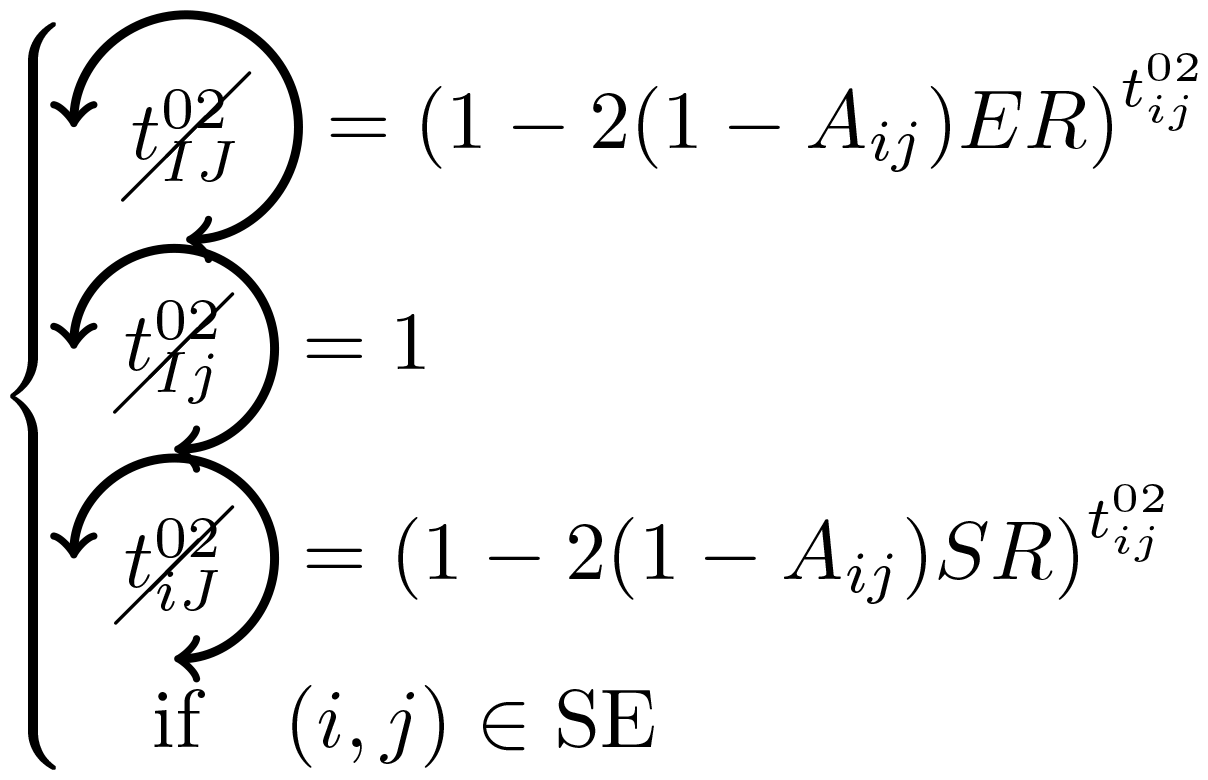
- *t*^11^ and *t*^12^ can contribute to FC if we have at least one pacemaker adjacent to a common neighbor and to *i* and *j* at one step:

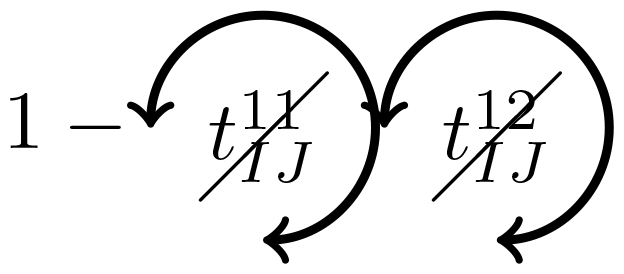

where

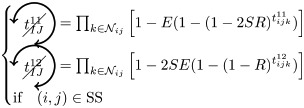
- *t*^13^ does not contribute directly to FC, if at least one triangle is used as a pacemaker then it necessarily imply that a triangle within *t*^01^ or *t*^02^ is also used as a pacemaker.

Assuming synchronization of the pair (*i*, *j*), the contribution of the different triangles is as follows:

- *t*^01^ can contribute to FC if at least one triangle is used as a pacemaker with excitation going from the common neighbor *k* to the pair (*i*, *j*). Additionally, in order to obtain the synchronization, (*i*, *k*, *j*) must not form a pacemaker of length 4 with another common neighbor. This condition is also valid for *t*^11^, when one pacemaker is attached to one the nodes (*i*, *j*):

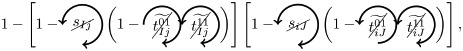

where 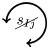 (resp. 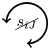) represents the probability of not having the pair (*i*, *j*) forming a pacemaker of length 4 when we have at least one pacemaker attached to *i* (resp. *j*) within the motifs *t*^01^ and *t*^11^,

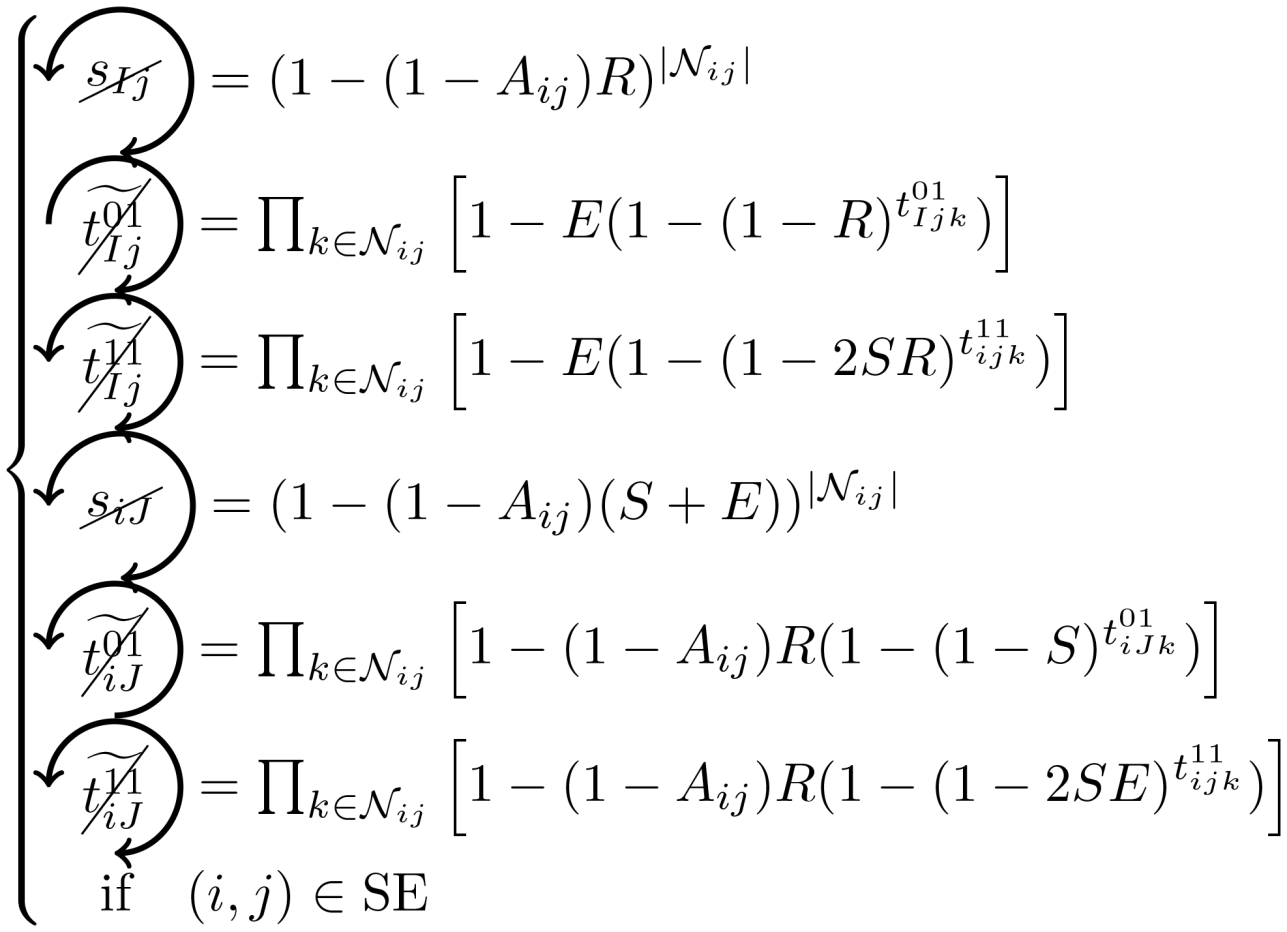
- if one *t*^11^ or *t*^12^ is used as a pacemaker and is not attached to *i* or *j*, then it can contribute to FC if the pair (*i*, *j*) are in the same initial state and no pacemaker is used in *t*^00^ and *t*^01^

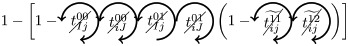

where

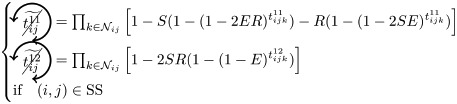

If the nodes *i* and *j* are in different initial states, then *t*^11^ can contribute to FC if no pacemaker is used in *t*^00^, *t*^01^, *t*^02^, and that the nodes *i* and *j* are not forming a pacemaker of length 4 with the common neighbor involved in *t*^11^ and one additional common neighbor

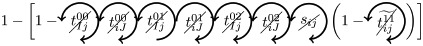

where

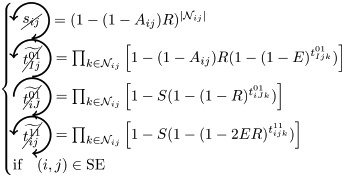

As for *t*^11^, *t*^12^ can contribute to FC if the nodes *i* and *j* are in different initial states, except that we can have pacemakers within *t*^00^, *t*^01^ or *t*^02^ motifs if *i* or *j* is already adjacent to the pacemaker in *t*^12^

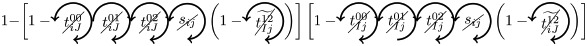

where

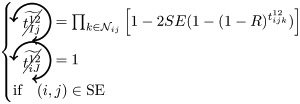

### Validation of the analytical prediction based on pacemakers

In order to validate our approach, we used synthetic toy graphs with a desired triangle motifs around a given pair of nodes. For each triangle motif, we then generated a random graph with 30 nodes. For triangle motifs involving common neighbors (ie, *t*^01^, *t*^02^, *t*^11^ and *t*^12^), we fixed the number of common neighbors to 6. Everything else was set randomly.

## Supporting information

S1 Fig Effect of network density on the analytical FC prediction for random graphs. Mean and standard deviation across network realizations of the correlation (first row) and mean difference (second row) between simulated and predicted FCs as a function of the number of nodes initially excited and the network density (shaded colored lines). Of note, the standard deviation is almost indistinguishable as it is very close to zero.
(EPS)

S2 Fig Validation of the analytical FC prediction with toy graphs. Set of toy networks (first column) with specific distributions of triangle motifs (second column) around a pair of nodes (in red, green nodes denotes their common neighbors), and the associated simulated and predicted FCs. The plots are function of the number of nodes initially excited and the analytical prediction (magenta, red, green and blue for the prediction from SC, TO—Eq 1, FC1—Eq 5 and FC2—Eq 6, respectively, black codes for the simulated FC).
(EPS)

S1 File Deterministic SER model (Matlab code). (M)

S2 File Analytical FC prediction in the deterministic SER model from FC1—Eq 5 and FC2—Eq 6 (Matlab code). (M)

S3 File Triangle motifs detection routine (Matlab code). (M)

S4 File Number of triangles around common neighbors, Eq 3 (Matlab code). (M)

## References

[1] Barabási AL, Pósfai M. Network science. Cambridge: Cambridge University Press; 2016 Available from: http://barabasi.com/networksciencebook/.

[2] Sporns O. Networks of the Brain. Cambridge, Massachusetts, United States: MIT Press; 2011.

[3] Zhou C, Zemanova L, Zamora-Lopez G, Hilgetag CC, Kurths J. Structure-function relationship in complex brain networks expressed by hierarchical synchronization. New Journal of Physics. 2007;9:178 doi: 10.1088/1367-2630/9/6/178

[4] Deco G, Jirsa V, Robinson PA, Breakspear M, Friston K. The dynamic brain: from spiking neurons to neural masses and cortical fields. PLoS Computational Biology. 2008;4:e1000092 doi: 10.1371/journal.pcbi.1000092 18769680 10.1371/journal.pcbi.1000092 PMC2519166

[5] Honey CJ, Sporns O, Cammoun L, Gigandet X, Thiran JP, Meuli R, et al Predicting human resting-state functional connectivity from structural connectivity. Proceedings of the National Academy of Sciences of the USA. 2009;106:2035–40. doi: 10.1073/pnas.0811168106 19188601 10.1073/pnas.0811168106 PMC2634800

[6] Petra R, Michael S, Anthony Randal M, Viktor K J. The Virtual Brain Integrates Computational Modeling and Multimodal Neuroimaging. Brain Connectivity. 2013;3(2):121–145. doi: 10.1089/brain.2012.0120 23442172 10.1089/brain.2012.0120 PMC3696923

[7] Joana C, Morten L K, Gustavo D. Exploring the network dynamics underlying brain activity at rest. Progress in Neurobiology. 2014;114:102–31. doi: 10.1016/j.pneurobio.2013.12.005 24389385 10.1016/j.pneurobio.2013.12.005

[8] Markram H, Muller E, Ramaswamy S, Reimann MW, Abdellah M, Sanchez CA, et al Reconstruction and Simulation of Neocortical Microcircuitry. Cell. 2015;163(2):456–492. doi: 10.1016/j.cell.2015.09.029 26451489 10.1016/j.cell.2015.09.029

[9] Deco G, Jirsa V, McIntosh AR, Sporns O, Kotter R. Key role of coupling, delay, and noise in resting brain fluctuations. Proceedings of the National Academy of Sciences of the USA. 2009;106:10302–7. doi: 10.1073/pnas.0901831106 19497858 10.1073/pnas.0901831106 PMC2690605

[10] Ghosh A, Rho Y, McIntosh AR, Kotter R, Jirsa V. Noise during rest enables the exploration of the brain’s dynamic repertoire. PLoS Computational Biology. 2008;4:e1000196 doi: 10.1371/journal.pcbi.1000196 18846206 10.1371/journal.pcbi.1000196 PMC2551736

[11] Cabral J, Hugues E, Sporns O, Deco G. Role of local network oscillations in resting-state functional connectivity. NeuroImage. 2011;57:130–9. doi: 10.1016/j.neuroimage.2011.04.010 21511044 10.1016/j.neuroimage.2011.04.010

[12] Haimovici A, Tagliazucchi E, Balenzuela P, Chialvo DR. Brain Organization into Resting State Networks Emerges at Criticality on a Model of the Human Connectome. Physical Review Letters. 2013;110:178101 doi: 10.1103/PhysRevLett.110.178101 23679783 10.1103/PhysRevLett.110.178101

[13] Messé A, Rudrauf D, Benali H, Marrelec G. Relating Structure and Function in the Human Brain: Relative Contributions of Anatomy, Stationary Dynamics, and Non-Stationarities. PLoS Computational Biology. 2014;10:e1003530 doi: 10.1371/journal.pcbi.1003530 24651524 10.1371/journal.pcbi.1003530 PMC3961181

[14] Messé A, Rudrauf D, Giron A, Marrelec G. Predicting functional connectivity from structural connectivity via computational models using MRI: An extensive comparison study. NeuroImage. 2015;111C:65–75. 10.1016/j.neuroimage.2015.02.001 25682944

[15] Sporns O, Chialvo DR, Kaiser M, Hilgetag CC. Organization, development and function of complex brain networks. Trends in Cognitive Sciences. 2004;8:418–25. doi: 10.1016/j.tics.2004.07.008 15350243 10.1016/j.tics.2004.07.008

[16] Bullmore E, Sporns O. Complex brain networks: graph theoretical analysis of structural and functional systems. Nature Reviews Neuroscience. 2009;10:186–98. doi: 10.1038/nrn2575 19190637 10.1038/nrn2575

[17] Goñi J, van den Heuvel MP, Avena-Koenigsberger A, Velez de Mendizabal N, Betzel RF, Griffa A, et al Resting-brain functional connectivity predicted by analytic measures of network communication. Proceedings of the National Academy of Sciences of the USA. 2014;111(2):833–838. doi: 10.1073/pnas.1315529111 24379387 10.1073/pnas.1315529111 PMC3896172

[18] Messé A, Benali H, Marrelec G. Relating structural and functional connectivity in MRI: A simple model for a complex brain. IEEE Transactions on Medical Imaging. 2015;34:27–37. doi: 10.1109/TMI.2014.2341732 25069111 10.1109/TMI.2014.2341732

[19] Saggio M, Ritter P, Jirsa V. Analytical Operations Relate Structural and Functional Connectivity in the Brain. PLoS One. 2016;11(8):e0157292 doi: 10.1371/journal.pone.0157292 27536987 10.1371/journal.pone.0157292 PMC4990451

[20] Greenberg JM, Hastings SP. Spatial Patterns for Discrete Models of Diffusion in Excitable Media. SIAM Journal on Applied Mathematics. 1978;34(3):515–523. doi: 10.1137/0134040

[21] Bak P. A forest-fire model and some thoughts on turbulence. Physics Letters A. 1990;147:297–300. doi: 10.1016/0375-9601(90)90451-S

[22] Drossel B, Schwabl F. Self-organized critical forest-fire model. Physical Review Letters. 1992;69:1629–1632. doi: 10.1103/PhysRevLett.69.1629 10046273 10.1103/PhysRevLett.69.1629

[23] Anderson R, May RM. Infectious Diseases of Humans: Dynamics and Control. Oxford: Oxford University Press; 1992.

[24] Grassly NC, Fraser C. Mathematical models of infectious disease transmission. Nature Reviews Microbiology. 2008;6(6):477–487. 18533288 10.1038/nrmicro1845 PMC7097581

[25] Furtado LS, Copelli M. Response of electrically coupled spiking neurons: A cellular automaton approach. Physical Review E (Statistical, Nonlinear, and Soft Matter Physics). 2006;73:011907+ doi: 10.1103/PhysRevE.73.011907 10.1103/PhysRevE.73.011907 16486185

[26] Kinouchi O, Copelli M. Optimal dynamical range of excitable networks at criticality. Nature Physics. 2006;2(5):348–351. doi: 10.1038/nphys289

[27] Müller-Linow M, Marr C, Hütt MT. Topology regulates the distribution pattern of excitations in excitable dynamics on graphs. Physical Review E. 2006;74:1–7. 10.1103/PhysRevE.74.016112 16907156

[28] Garcia GC, Lesne A, Hütt MT, Hilgetag CC. Building blocks of self-sustained activity in a simple deterministic model of excitable neural networks. Frontiers in Computational Neuroscience. 2012;6:50 doi: 10.3389/fncom.2012.00050 22888317 10.3389/fncom.2012.00050 PMC3412572

[29] Hütt MT, Hilgetag CC, Kaiser M. Network-guided pattern formation of neural dynamics. Philosophical Transactions of the Royal Society of London Series B, Biological Sciences. 2014;369:20130522 doi: 10.1098/rstb.2013.0522 25180302 10.1098/rstb.2013.0522 PMC4150299

[30] Messé A, Hütt MT, König P, Hilgetag C. A closer look at the apparent correlation of structural and functional connectivity in excitable neural networks. Scientific Reports. 2015;5:7870 doi: 10.1038/srep07870 25598302 10.1038/srep07870 PMC4297952

[31] Carvunis AR, Latapy M, Lesne A, Magnien C, Pezard L. Dynamics of three-state excitable units on Poisson vs. power-law random networks. Physica A: Statistical Mechanics and its Applications. 2006;367:595–612. doi: 10.1016/j.physa.2005.12.056

[32] Ravasz E, Somera AL, Mongru DA, Oltvai ZN, Barabási AL. Hierarchical Organization of Modularity in Metabolic Networks. Science. 2002;297(5586):1551–1555. doi: 10.1126/science.1073374 12202830 10.1126/science.1073374

[33] Pastor-Satorras R, Castellano C, Van Mieghem P, Vespignani A. Epidemic processes in complex networks. Reviews of Modern Physics. 2015;87:925–979. doi: 10.1103/RevModPhys.87.925

[34] Vladimirov N, Tu Y, Traub R. Shortest Loops are Pacemakers in Random Networks of Electrically Coupled Axons. Frontiers in Computational Neuroscience. 2012;6:17 doi: 10.3389/fncom.2012.00017 22514532 10.3389/fncom.2012.00017 PMC3324298

[35] Moretti P, Muñoz M. Griffiths phases and the stretching of criticality in brain networks. Nature Communications. 2013;4 doi: 10.1038/ncomms3521 24088740 10.1038/ncomms3521

[36] Ódor G. Critical dynamics on a large human Open Connectome network. Physical Review E. 2016;94:062411 doi: 10.1103/PhysRevE.94.062411 28085398 10.1103/PhysRevE.94.062411

[37] de Haan W, van Straaten ECW, Gouw AA, Stam CJ. Altering neuronal excitability to preserve network connectivity in a computational model of Alzheimer’s disease. PLOS Computational Biology. 2017;13(9):1–23. doi: 10.1371/journal.pcbi.1005707 10.1371/journal.pcbi.1005707 PMC5627940 28938009

[38] Müller-Linow M, Hilgetag CC, Hütt MT. Organization of Excitable Dynamics in Hierarchical Biological Networks. PLoS Computational Biology. 2008;4:e1000190 doi: 10.1371/journal.pcbi.1000190 18818769 10.1371/journal.pcbi.1000190 PMC2542420

[39] Erdős P, Rényi A. On the evolution of random graphs. Publications of the Mathematical Institute of the Hungarian Academy of Sciences. 1960;5:17–61.

[40] Barabási AL, Albert R. Emergence of scaling in random networks. Science. 1999;286:15551–15555. 10.1126/science.286.5439.509 10521342

[41] Hagberg A, Schult D, Swart P. Exploring network structure, dynamics, and function using NetworkX. In: Proceedings of the 7th Python in Science Conference (SciPy2008). Pasadena, United States; 2008. p. 11–15.

[42] Kamada T, Kawai S. An algorithm for drawing general undirected graphs. Information Processing Letters. 1989;31:7–15. doi: 10.1016/0020-0190(89)90102-6

[43] Rubinov M, Sporns O. Complex network measures of brain connectivity: Uses and interpretations. NeuroImage. 2010;52:1059–69. doi: 10.1016/j.neuroimage.2009.10.003 19819337 10.1016/j.neuroimage.2009.10.003

